# Age-Associated Plasma Protein Signatures and Proteomic Age Estimation by Data-Independent Acquisition Mass Spectrometry in a Russian Cross-Sectional Cohort

**DOI:** 10.3390/ijms27198707

**Published:** 2026-09-29

**Authors:** Mikhail S. Arbatskiy, Dmitriy E. Balandin, Svetlana E. Novikova, Nikita E. Vavilov, Valery A. Maiorov, Alexey V. Churov

**Affiliations:** 1 Institute on Aging Research, Russian Clinical Research Center of Gerontology, Pirogov Russian National Research Medical University, Ministry of Healthcare of the Russian Federation, 129226 Moscow, Russiaachurou@yandex.ru (A.V.C.); 2 Institute of Biomedical Chemistry, 119121 Moscow, Russia; novikova.s.e3101@gmail.com (S.E.N.); vamaiorov23@gmail.com (V.A.M.)

**Keywords:** chronological-age prediction, age-associated plasma proteins, data-independent acquisition mass spectrometry, DIA-MS, plasma proteomics, gradient boosting, batch effects, proof-of-concept

## Abstract

Chronological age can be estimated from molecular profiles; however, predictive accuracy alone does not establish a biologically validated ageing clock. We therefore evaluated whether plasma protein intensities measured by data-independent acquisition mass spectrometry (DIA-MS) support chronological-age estimation in a cross-sectional cohort from Moscow, Russia, while explicitly examining acquisition-order and MS-batch confounding. Plasma samples from 350 adult participants aged 19.8 to 99.1 years were analyzed as single-shot, single-injection DIA-MS acquisitions on a Q Exactive HFX platform. The final matrix comprised 222 protein groups and was generated with DIA-NN version 1.8.1 using a two-pass predicted-library-assisted workflow, cross-run precursor-level normalization, normalized MaxLFQ-like protein–group quantification, and 1% precursor and protein–group false-discovery-rate thresholds. Descriptive associations were assessed using Spearman rank correlation with Bonferroni correction. Feature selection and missing-value imputation were confined to each training subset. Generalization was assessed primarily by holding out complete MS batches, with repeated, nested, and random five-fold analyses retained as internal comparators. Of the 222 proteins, 148 were associated with chronological age at the nominal threshold *p* < 0.05, and 98 remained significant after Bonferroni correction. The strongest descriptive associations involved fibrinogen beta chain (FGB; rs = 0.590), SERPINA3 (rs = 0.560), ITIH4 (rs = 0.542), complement C5 (rs = 0.538), and ceruloplasmin (rs = 0.518). In internal random-split analyses, a 50-feature Gradient Boosting strategy yielded MAE = 8.90 ± 1.06 years in repeated five-fold cross-validation and MAE = 9.10 ± 1.10 years in nested cross-validation. However, when complete MS batches were held out, aggregate MAE increased to 10.56 years (R^2^ = 0.554) using a batch-stratified assignment and 11.87 years (R^2^ = 0.461) using GroupKFold. These batch-held-out results constitute the primary estimate of generalization from the present dataset. To assess the trade-off between error and assay complexity, strategies using 5, 8, 10, 12, 15, 20, 30, and 50 proteins selected within each training fold were compared. The 8-feature strategy yielded MAE = 11.83 years in repeated random cross-validation, whereas the 50-feature strategy yielded MAE = 8.90 years. These estimates concern feature-number strategies, not fixed panels. The post hoc eight-protein list is reported only as an exploratory candidate set for future independent validation. Acquisition order was associated with chronological age (rs = 0.336, *p* = 1.03 × 10^−10^), and age and sex distributions differed strongly across 27 MS batches. Order alone predicted age with MAE = 3.98 years in a negative-control model, demonstrating that age information was encoded in the acquisition design. Accordingly, the present findings support only a proof-of-concept for plasma-proteomic prediction of chronological age within this dataset; they do not establish a batch-independent model, a clinical tool, or a biologically validated ageing clock.

## 1. Introduction

Human ageing is a complex, multilevel process in which molecular, physiological, and functional changes develop at individual rates that vary across people. For this reason, chronological age provides only an approximate reflection of an organism’s biological state: individuals of the same calendar age may differ substantially in functional status, disease burden, severity of frailty, and risk of death [1]. This observation has served as the starting point for the development of so-called “molecular ageing clocks”: models that estimate the age-associated biological state of the organism from molecular or clinical features.

The most extensively studied systems of this kind are based on DNA methylation patterns or clinical biochemical parameters. Horvath’s clock demonstrated that age can be estimated from methylation profiles of CpG sites across different tissues [2]. The Klemera–Doubal method and the PhenoAge model extended this logic to clinical biomarkers and mortality-associated phenotypes [3,4]. More recent epigenetic clocks, in particular GrimAge, incorporate molecular surrogate markers related to smoking, plasma proteins, and mortality risk [5]. It is important to emphasize a key distinction shared by all these approaches: successful prediction of chronological age does not, in itself, mean that a model measures biological age. Clinical and biological relevance requires separate evidence; it must be shown that accelerated biological ageing identified by the model is genuinely associated with outcomes such as mortality, functional decline, disease development, or individual ageing rates in longitudinal observations [6,7].

Plasma proteomics offers an independent and informative perspective on ageing processes: plasma proteins reflect the state of inflammatory signalling pathways, the blood coagulation system, the complement cascade, tissue remodelling, metabolism, and organ-specific physiology. Large-scale plasma proteomics has already become an important tool in population biobank studies [8]. Studies using affinity-based platforms such as SOMAscan and Olink have shown that hundreds to thousands of plasma proteins change with age, that proteomic ageing may follow a nonlinear trajectory across the lifespan, and that proteomic clocks can be associated with disease and mortality [9,10,11]. At the same time, affinity-based platforms measure predefined sets of proteins and are inherently dependent on the composition of the reagent panels used. Mass-spectrometry-based proteomics offers a more open strategy for plasma profiling that is not restricted to a preselected list of targets. In particular, data-independent acquisition mass spectrometry (DIA-MS), combined with data-processing software such as DIA-NN, enables reproducible quantitative assessment of proteins in large sample series [12].

The possibility of predicting age from plasma proteomics using mass spectrometry has been investigated previously. Salignon and colleagues used proteomic and small RNA data from human plasma and showed that protein-based models can predict age, while the integration of protein and miRNA data improves prediction accuracy [13]. Against this background, the specific contribution of the present study is the evaluation of an exclusively DIA-MS-based analytical workflow in a Russian cohort of 350 participants, using a standardized panel of 222 plasma proteins and a validation scheme that confines feature selection to the training data.

The present study addresses an early analytical question: whether plasma protein intensities obtained by DIA-MS in this cohort contain an age-associated signal that supports chronological-age prediction, and how that signal behaves under leakage-aware random validation and complete-batch holdout. Approaches to estimating biological age from blood and urine biomarkers have been described previously [14], but biological-age interpretation additionally requires validation against outcomes such as mortality, functional decline, disease onset, or longitudinal ageing trajectories. Such validation was not available here.

During model development, cross-validated feature-number strategies were distinguished from fixed panels. A 50-feature strategy served as the high-dimensional research benchmark, while reduced strategies with 5, 8, 10, 12, 15, 20, and 30 fold-selected proteins were evaluated exploratorily. The specific eight-protein list in Supplementary Table S9 was selected from the full cohort after model comparison and has not undergone independent fixed-panel validation.

In summary, this study analyzed 350 plasma samples from adults aged 19.8 to 99.1 years, identified descriptive age-associated proteins, compared leakage-aware modelling strategies, and examined sensitivity to sex, age, acquisition order, and MS batch. Because age was strongly structured by the acquisition design and no external cohort was available, the results are framed as a proof-of-concept for chronological-age prediction rather than as a validated biological-age clock.

## 2. Results

### 2.1. Cohort Characteristics and DIA-MS Proteomic Coverage

**Figure 1:**
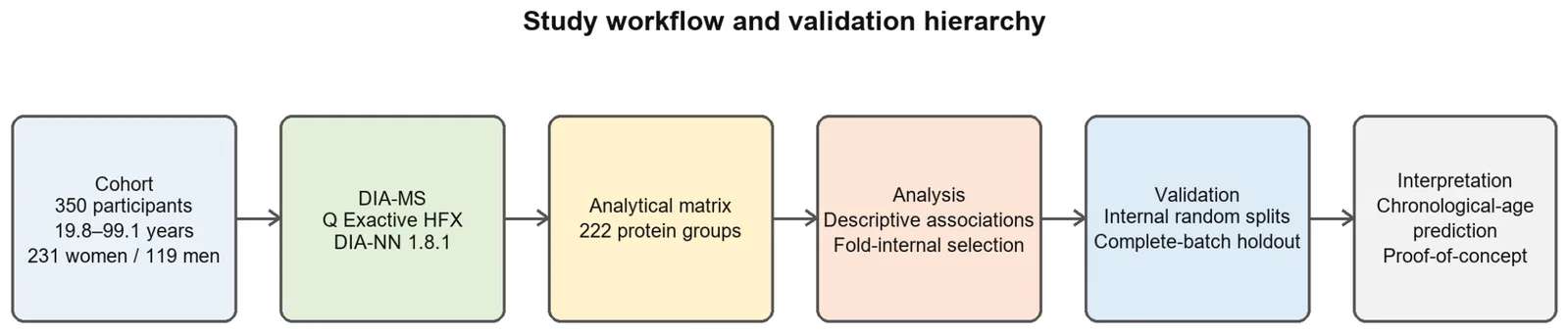
Study workflow and validation hierarchy for chronological-age prediction from plasma DIA-MS data. Random-split analyses were used for internal comparison, whereas complete-batch holdout provided the primary estimate of generalization.

The overall study design is presented in Figure 1. The analysis included 350 adult participants aged 19.8 to 99.1 years. Women accounted for 66.0% of the cohort (*n* = 231), and men for 34.0% (*n* = 119). The sex imbalance became more pronounced in the older age groups: among participants aged 60–74 years, there were 68 women and only 15 men, while in the group aged 75 years and older there were 54 women and 15 men. This imbalance is important for the appropriate interpretation of both the predictive performance of the model and the composition of the identified age-associated protein markers, and is discussed in more detail in Section 3.6.

DIA-MS profiling of plasma generated a matrix of semi-quantitative intensities comprising 222 protein groups across 350 samples. This panel reflects the coverage achievable using a standardized 30-min workflow without prior immunodepletion of highly abundant proteins and is not intended to provide exhaustive representation of low-abundance regulatory components of the plasma proteome.

### 2.2. Descriptive Age-Associated Plasma Proteins

**Figure 2:**
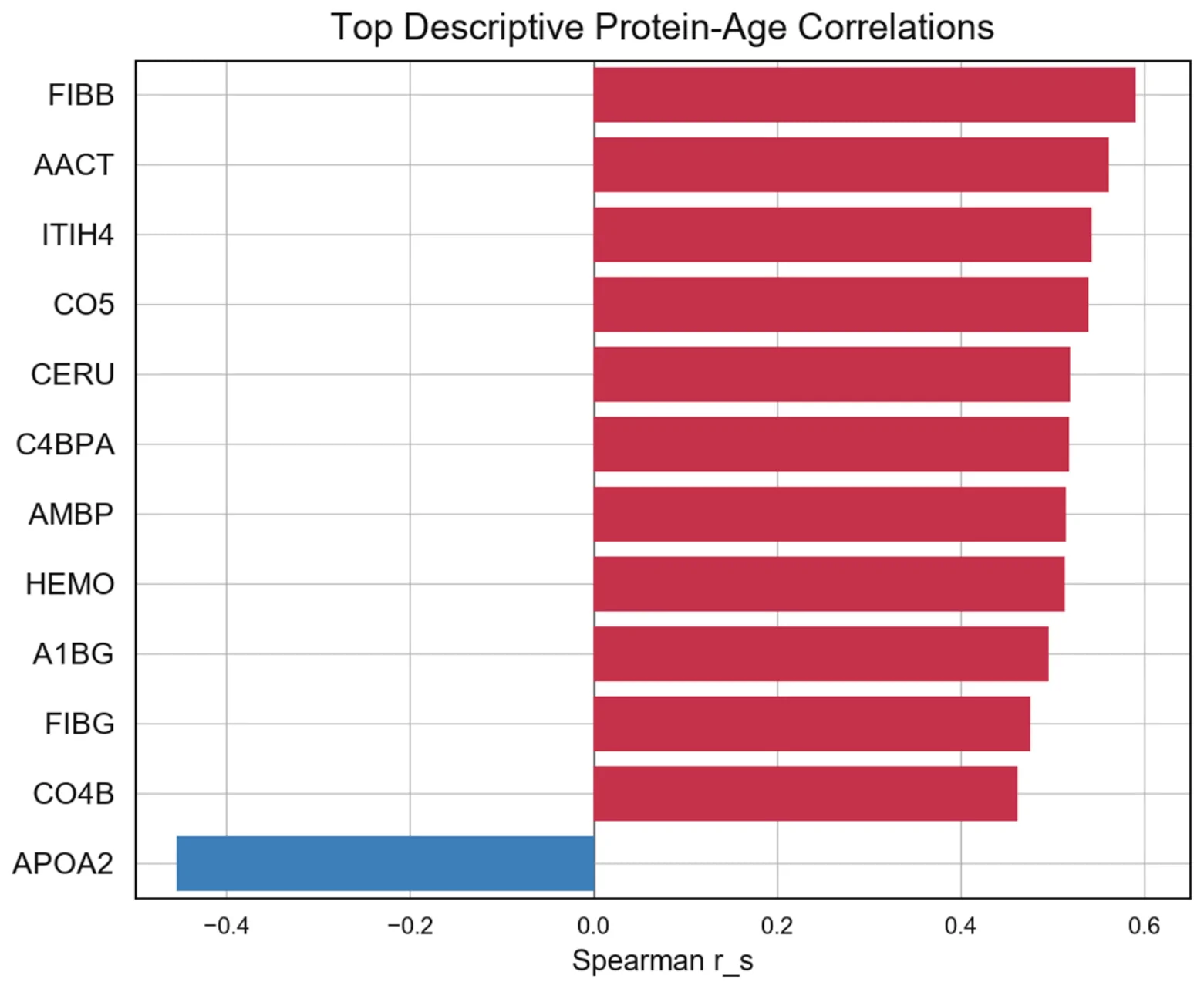
The strongest descriptive correlations of proteins with age. Full-sample correlations are presented for descriptive purposes only and were not used as leakage-free feature selection for the predictive model.

Spearman correlation analysis performed across the entire cohort revealed a broad association between plasma protein intensities and chronological age (Figure 2). Of the 222 proteins, 148 (66.7%) showed a statistically significant correlation at the nominal significance level of *p* < 0.05; of these, 98 proteins (44.1%) remained significant after Bonferroni correction for multiple testing (adjusted threshold *p* < 2.25 × 10^−4^). Importantly, these findings are descriptive in nature: they reflect the age-associated structure of the proteome in this cohort but were not used as a feature-selection criterion for building the predictive models. To prevent leakage of validation data, feature selection was performed exclusively within the training subsets (see Section 4.6.2).

The strongest positive correlations with age were observed for the following proteins: fibrinogen beta chain (FGB; rs = 0.590), alpha-1-antichymotrypsin (SERPINA3; rs = 0.560), inter-alpha-trypsin inhibitor heavy chain H4 (ITIH4; rs = 0.542), complement component C5 (rs = 0.538), ceruloplasmin (CP; rs = 0.518), C4b-binding protein alpha chain (C4BPA; rs = 0.518), protein AMBP (alpha-1-microglobulin/bikunin precursor; AMBP; rs = 0.513), hemopexin (HPX; rs = 0.513), alpha-1B-glycoprotein (A1BG; rs = 0.495), and fibrinogen gamma chain (FGG; rs = 0.475). The ten strongest descriptive markers, together with their biological classification, are presented in Table 1.

From a thematic perspective, the leading markers are distributed across several biological clusters: proteins of the blood coagulation system and the acute-phase response (FGB, FGG, SERPINA3, ITIH4), components and regulators of the complement system (C5, C4BPA), and proteins involved in oxidative stress, metal homeostasis, and heme metabolism (ceruloplasmin, hemopexin). The biological interpretation of these associations is discussed in detail in Section 3.2.

To assess the influence of cohort sex composition, an additional correlation analysis was performed using residuals adjusted for reported sex. The strongest sex-adjusted associations were observed for C1 inhibitor (rs = 0.539), SERPINA3 (rs = 0.520), ITIH4 (rs = 0.514), zinc-alpha-2-glycoprotein (ZA2G; rs = 0.512), C5 (rs = 0.504), and FGB (rs = 0.494). These results show that age associations remain detectable after adjustment for sex alone; however, they do not establish independence from acquisition order or MS batch, both of which were strongly structured by age and sex in the augmented metadata (Section 2.6).

### 2.3. Internal Leakage-Aware Random-Split and Nested Validation

When developing predictive models, a key requirement was to prevent leakage of validation data into the feature-selection stage. To this end, proteins were ranked according to their association with age strictly within each training subset at every cross-validation split, without any use of data from the corresponding validation fold.

**Figure 3:**
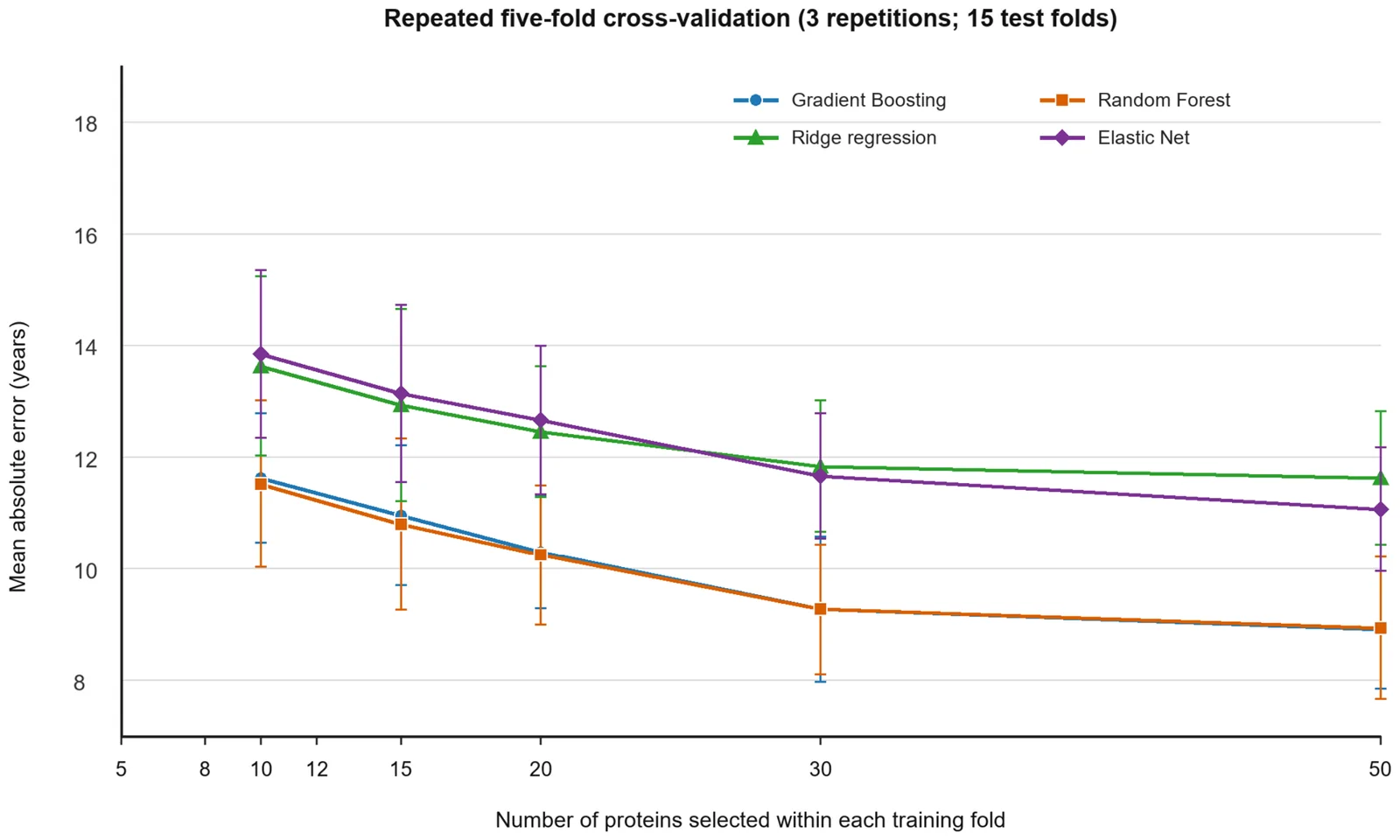
Internal model comparison in repeated five-fold cross-validation (CV). The *x*-axis gives the number of proteins selected within each training fold; the *y*-axis gives mean absolute error (MAE, years). Points show the mean across 15 test folds (five folds × three repetitions), and error bars show the standard deviation. Full model names are provided in the legend. CV, cross-validation.

In repeated five-fold cross-validation, comprising three repetitions with independent split configurations, the best performance among all tested settings was achieved by a Gradient Boosting model using 50 fold-selected proteins: the arithmetic mean of the 15 fold-level MAEs was 8.90 ± 1.06 years, and the mean coefficient of determination was R^2^ = 0.698 ± 0.078. A Random Forest model with the same panel size showed comparable repeated-CV performance (MAE = 8.93 ± 1.27 years, R^2^ = 0.691 ± 0.083). The latter value is a separate algorithm result and should not be confused with the aggregate MAE of 8.93 years obtained from the single five-fold Gradient Boosting out-of-fold prediction vector used for subgroup summaries. Linear models and configurations with fewer features generally demonstrated lower predictive accuracy. A comparison of all algorithms and panel sizes is presented in Figure 3.

Nested cross-validation was additionally used to account for uncertainty in algorithm and feature-count selection. The outer loop consisted of five splits, while three-fold inner cross-validation selected the algorithm and feature number. Nested cross-validation yielded MAE = 9.10 ± 1.10 years and R^2^ = 0.680 ± 0.086. This is the most conservative internal random-split estimate, but it does not protect against acquisition-design confounding because samples from the same run-order and batch structure can still occur in both training and test folds. Complete-batch holdout in Section 2.6 therefore provides the primary estimate of generalization from this dataset.

**Figure 4:**
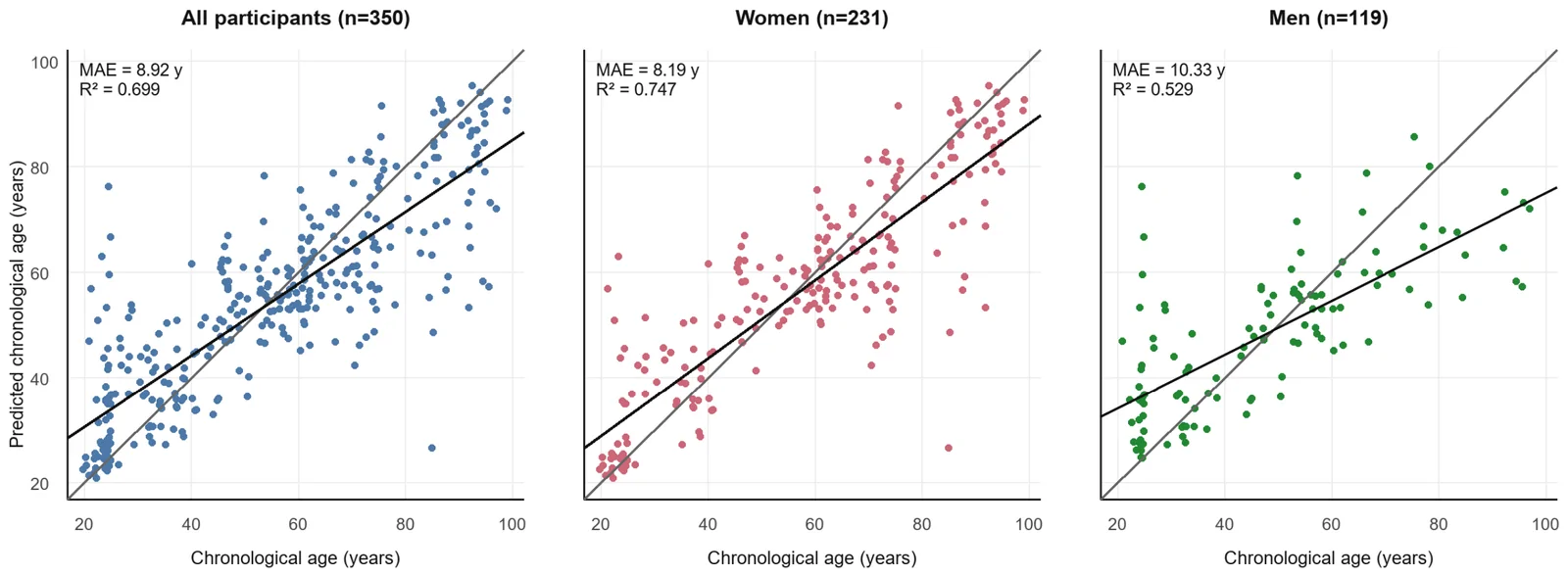
Random five-fold out-of-fold (OOF) predictions for all participants, women, and men. Dashed lines indicate identity (predicted age = chronological age); solid lines indicate fitted linear trends. Each panel reports *n*, MAE, and R^2^. Sex-specific results are exploratory because of cohort imbalance and are not evidence of biological differences in ageing.

Out-of-fold predictions from the predefined random five-fold Gradient Boosting analysis are shown for the full cohort, women, and men in Figure 4. The combined analysis yielded MAE = 8.93 years and R^2^ = 0.704; subgroup estimates were MAE = 8.19 years and R^2^ = 0.747 for women and MAE = 10.37 years and R^2^ = 0.546 for men. These subgroup comparisons are exploratory because the cohort contained 231 women and 119 men, with only 15 men in each of the two oldest age groups. The regression-to-the-mean pattern is visible in all panels and should not be interpreted as a sex-specific biological difference.

### 2.4. Reduced Feature-Number Strategies and Exploratory Candidate Panel

To evaluate the trade-off between model complexity and prediction error, strategies using 5, 8, 10, 12, 15, 20, 30, and 50 features were tested. In each cross-validation fold, proteins were ranked using only the corresponding training subset; consequently, the selected proteins could differ between folds. The reported metrics therefore quantify feature-number strategies and do not validate a single fixed panel.

The results for gradient boosting are presented in Table 2. As the number of fold-selected features increased, prediction accuracy improved: MAE values were 13.38, 11.83, 11.62, 11.27, 10.96, 10.30, 9.27, and 8.90 years for the 5-, 8-, 10-, 12-, 15-, 20-, 30-, and 50-feature strategies, respectively. These values must not be attributed to the post hoc fixed candidate panels listed in the Supplementary Information.

Across the evaluated feature-set sizes, internal random-split error decreased from MAE = 13.38 years with 5 fold-selected features to MAE = 8.90 years with 50 fold-selected features. This trend describes strategies in which proteins were reselected within each training split; it does not validate any single fixed panel. Because random splitting does not separate the acquisition design, these values are internal comparison metrics rather than the primary generalization estimate.

Thus, the 50-feature strategy provides the best cross-validated accuracy within the scope of this analysis and serves as a research benchmark. The 8-feature strategy retains an age-associated signal at higher error and motivates exploration of smaller assays. The specific fixed panel FIBB, AACT, ITIH4, CO5, CERU, C4BPA, AMBP, and HEMO was derived post hoc from the full cohort. It exactly matched the eight proteins selected within the training data in 2 of 5 folds; each of the other three folds shared 7 of 8 proteins with the fixed list. This overlap supports stability as a candidate set but does not constitute an unbiased performance validation of the fixed panel, which therefore remains exploratory and requires evaluation in an independent dataset.

### 2.5. Sensitivity by Sex and Age

Model performance in stratified subgroups was assessed using a single predefined five-fold out-of-fold prediction vector from the 50-feature Gradient Boosting analysis. The aggregate MAE of this vector was 8.93 years; it was used only for subgroup summaries and is distinct from the repeated-CV mean of 8.90 ± 1.06 years reported in Section 2.3. Subgroup summaries emphasize *n*, MAE, mean prediction residual (predicted minus chronological age), and nonparametric bootstrap 95% confidence intervals based on 10,000 resamples. R^2^ is reported for the overall cohort and sex strata but not for narrow age strata, where the restricted age range makes it unstable and difficult to interpret.

For the 50-feature Gradient Boosting analysis, women had an MAE of 8.19 years (95% CI, 7.15–9.27; *n* = 231) and a mean prediction residual of −1.03 years (95% CI, −2.57 to 0.43); men had an MAE of 10.37 years (95% CI, 8.76–12.09; *n* = 119) and a mean residual of 0.95 years (95% CI, −1.54 to 3.49). Overall sex-stratum R^2^ values were 0.747 and 0.546, respectively. These differences should not be interpreted as intrinsic sex differences in biological ageing; they are plausibly influenced by cohort and acquisition structure.

Across age groups, the mean prediction residual shifted from +8.95 years (95% CI, 6.14–12.05; *n* = 64) at 18–24 years to −10.29 years (95% CI, −13.40 to −7.24; *n* = 69) at ≥75 years, consistent with regression to the mean. Men aged ≥75 years had an MAE of 19.10 years (95% CI, 14.65–23.45) and a mean residual of −17.65 years (95% CI, −23.11 to −11.64), but this subgroup comprised only 15 participants. Men aged 60–74 years likewise comprised 15 participants. These estimates are exploratory, have wide uncertainty, and do not support biological or clinical conclusions.

These exploratory subgroup results support two conclusions. First, prediction uncertainty is substantial at the individual level and varies across the age distribution. Second, the observed difference between women and men cannot be attributed to sex-specific ageing biology because the older male strata were small and the acquisition design was confounded with age and sex. Independent, balanced validation is required before subgroup claims can be made.

### 2.6. Primary Complete-Batch-Holdout Assessment and Acquisition-Design Sensitivity

The analytical matrix included the acquisition order (1–350) and membership in one of 27 MS batches for each participant. Chronological age was positively associated with acquisition order (Spearman rs = 0.336, *p* = 1.03 × 10^−10^) and differed strongly across batches (Kruskal–Wallis H = 323.38, *p* = 4.33 × 10^−53^). Sex composition also differed across batches (χ^2^ = 345.92, 26 degrees of freedom, *p* = 1.23 × 10^−57^). These findings show that the acquisition series was not randomized or balanced with respect to the demographic variables used in the age-prediction analysis.

As negative controls, metadata-only Gradient Boosting models were evaluated using the same random five-fold split. Acquisition order alone predicted chronological age with an aggregate MAE of 3.98 years (R^2^ = 0.924), MS batch alone with an MAE of 5.11 years (R^2^ = 0.898), and the combination of order, batch, and sex with an MAE of 3.85 years (R^2^ = 0.942). These models are not biological estimators; rather, they demonstrate that chronological age is strongly encoded in the acquisition design.

**Figure 5:**
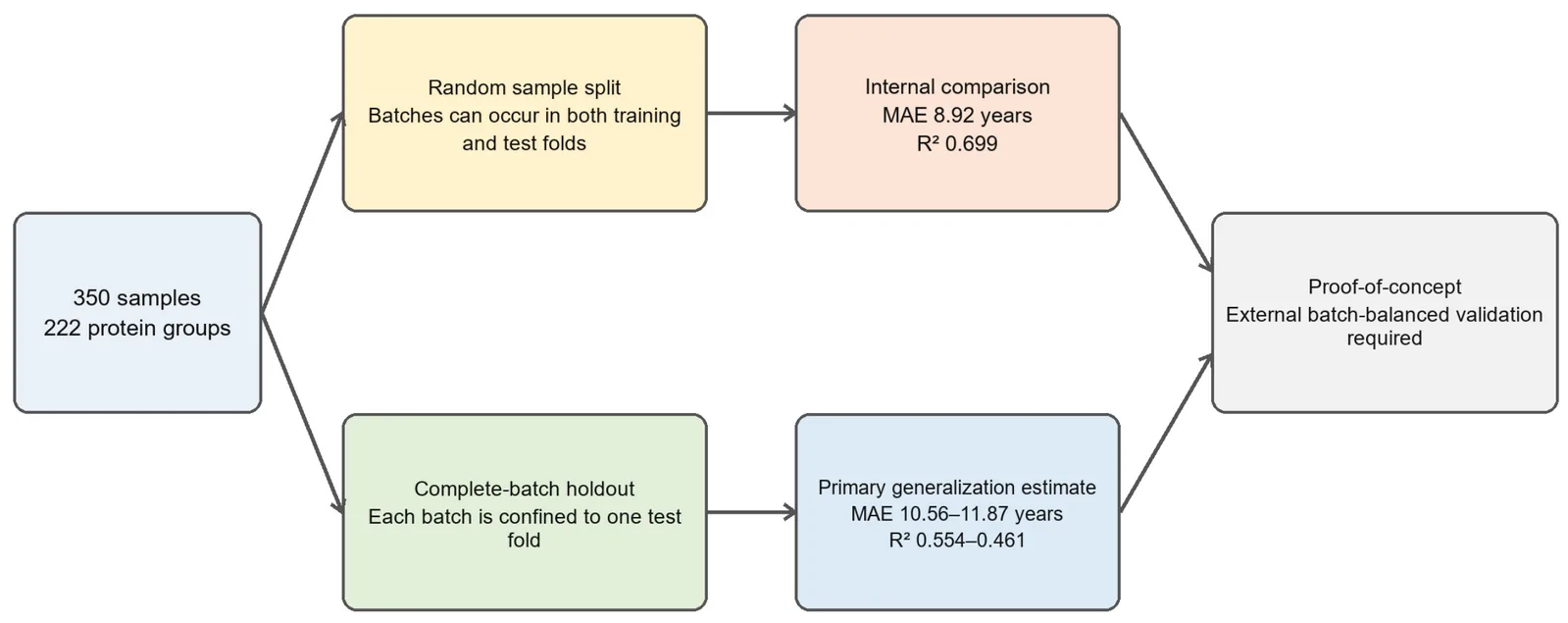
Distinction between internal random-split analysis and complete-batch-holdout validation. Random splitting can place samples from the same acquisition structure in training and test folds; complete-batch holdout keeps every MS batch within a single fold and therefore provides the primary generalization estimate for this dataset.

In a matched single random five-fold analysis, the 50-feature protein strategy yielded aggregate MAE = 8.92 years and R^2^ = 0.699. When complete MS batches were held out, aggregate MAE increased to 10.56 years (R^2^ = 0.554) with a batch-stratified assignment and 11.87 years (R^2^ = 0.461) with standard GroupKFold. These two complete-batch-holdout estimates are the primary performance results because they more directly test transfer to independently acquired batches. Residuals from the random-split model remained associated with order (rs = −0.202, *p* = 1.46 × 10^−4^) and differed across batches (H = 117.66, *p* = 1.27 × 10^−13^). PCA likewise showed strong batch structure (batch η^2^ = 0.850 for PC1 and 0.876 for PC2; PC2 versus order rs = 0.861). Validation estimates are summarized in Table 3, the two split strategies are shown in Figure 5, and fold-level results are provided in Supplementary Tables S12–S14.

An exploratory partial-rank analysis adjusting simultaneously for sex, acquisition order, and batch yielded no protein passing the Bonferroni threshold. Because age was strongly collinear with order and batch, conventional post hoc correction could remove genuine age-associated variation together with technical variation and could not reconstruct a randomized design. No additional batch correction was therefore applied to the final matrix. Instead, the analysis reports design diagnostics, metadata-only negative controls, PCA, residual tests, and complete-batch holdout. This conservative approach follows recommendations that batch correction in large proteomic datasets must be evaluated against study design and preservation of biological signal [15,16]. The sensitivity analyses do not show that the proteomic signal is absent; they show that its biological and technical components cannot be cleanly separated in this cohort.

## 3. Discussion

### 3.1. Chronological-Age Prediction and Control of Methodological Biases

The present study shows that plasma protein intensities measured by DIA-MS contain a signal associated with chronological age in this cohort. Descriptive analysis identified 148 nominally age-associated proteins and 98 proteins after Bonferroni correction. However, acquisition order and MS batch also encoded age strongly. Accordingly, the primary complete-batch-holdout analyses yielded MAE = 10.56–11.87 years, whereas the lower errors of 8.90 years in repeated random cross-validation and 9.10 years in nested random cross-validation should be interpreted only as internal comparisons within the same acquisition design.

Feature selection was confined to each training subset, preventing validation samples from contributing to protein ranking or imputation. This addresses feature-selection leakage, but it does not address a different problem: random folds can still share acquisition-design structure. The distinction between leakage control and batch-independent generalization is therefore central to interpretation of the model.

Nested cross-validation accounted for algorithm and feature-count selection within the random-split framework. It is therefore a rigorous internal model-selection analysis, but it is not the primary generalization estimate in the presence of batch confounding. Complete-batch holdout provides the more relevant estimate for transfer across acquisition batches.

Acquisition metadata identified substantial design confounding: age and sex were not independent of run order or MS batch, and metadata-only models predicted age with low error. The decrease in protein-model performance under complete-batch holdout, together with residual and PCA diagnostics, indicates that random-split estimates are optimistic for batch-independent deployment. The present design cannot determine how much of the predictive signal is biological and how much is technical.

Taken together, the findings support a conservative conclusion: the workflow provides proof-of-concept evidence for predicting chronological age from the 222 plasma protein groups detectable under this DIA-MS protocol. It does not establish a comprehensive ageing signature, a clinically deployable model, or a biologically validated ageing clock.

### 3.2. Biological Interpretation of Age-Associated Proteins

The strongest descriptive markers fit into a biologically plausible picture of molecular changes associated with ageing. The fibrinogen beta (FGB) and gamma (FGG) chains reflect the state of the blood coagulation system and the acute-phase response. An age-related increase in fibrinogen levels has been described in numerous population-based studies and has been linked to both chronic subclinical inflammation and increasing cardiovascular risk. Alpha-1-antichymotrypsin (SERPINA3), alpha-1-microglobulin/bikunin precursor (AMBP), alpha-1B-glycoprotein (A1BG), and inter-alpha-trypsin inhibitor heavy chain H4 (ITIH4) are involved in the acute-phase and inflammatory responses, as well as in the regulation of the extracellular matrix. Their coordinated increase with age is consistent with the concept of chronic low-grade systemic inflammation accompanying ageing, commonly referred to as “inflammageing” [17]. Components and regulators of the complement system, C5 and C4BPA, indicate activation or remodelling of innate immune pathways. Finally, ceruloplasmin and hemopexin reflect the status of antioxidant defence systems, metal ion metabolism, and haem clearance.

At the same time, an important caveat should be made. Because detailed clinical covariates for the participants—including body mass index, smoking status, medication use, C-reactive protein levels, and the presence of comorbidities—were not available in the present study, the observed associations cannot be unequivocally attributed to ageing itself. Many of the strongest markers are not specific molecular clocks of cellular ageing, but rather broad proteins of the systemic host response. Their association with chronological age may be partially or even entirely explained by increasing subclinical inflammation, changes in body composition, accumulation of comorbid conditions, or medication use among older participants. This represents a key limitation when interpreting the biological significance of the identified markers.

Additional analysis adjusted for sex showed that the age-associated signal generally persisted when sex alone was included as a covariate, although the ranking of individual proteins changed. In particular, C1 inhibitor and zinc-alpha-2-glycoprotein moved up in the ranking, whereas several coagulation-related proteins shifted lower. This analysis addresses one demographic imbalance but cannot demonstrate that the signature is independent of acquisition order or MS batch. Because age and sex were both strongly structured across the acquisition series, the observed protein–age associations may contain inseparable biological and technical components. Accordingly, the present marker list should not be presented as a universal, sex- and batch-independent signature of ageing.

### 3.3. Interpretation of Proteomics-Based Chronological-Age Estimates

Because the model was trained to predict chronological age from plasma protein intensities, its output is most appropriately described as a proteomics-based estimate of chronological age. The difference between predicted and observed chronological age may be reported descriptively as a prediction residual, but it cannot be interpreted as accelerated or decelerated biological ageing without independent association with clinically meaningful outcomes.

The present analysis supports a distinction between a high-dimensional research benchmark and exploratory work toward a reduced assay. The 50-feature strategy provided the highest cross-validated accuracy. The 8-feature strategy yielded an MAE of 11.83 years when the eight proteins were reselected within each training fold. The specific fixed eight-protein panel listed in Supplementary Table S9 was selected using the full cohort and was not evaluated in independent data; it is therefore a candidate panel for future validation rather than a validated applied configuration.

The model output is an experimental estimate of chronological age derived from DIA-MS protein intensities, not a diagnostic conclusion about an individual’s biological age. Translation beyond proof-of-concept requires external validation, a prospectively randomized or balanced acquisition design, standardized quality control, and evidence of association with clinically meaningful outcomes.

Caution is also warranted when comparing this model with established ageing clocks. The Horvath epigenetic clock, GrimAge, PhenoAge, and systems based on the Klemera–Doubal method were developed not merely as age-prediction tools; many of them have undergone validation with respect to mortality, healthspan, clinical phenotypes, or disease outcomes [2,3,4,5,6]. The present model provides an estimate of proteomic age in a single cross-sectional cohort without clinical validation. Therefore, it is appropriate to compare it with related studies only in terms of prediction error, while explicitly stating that its biological and clinical validity have not yet been established.

### 3.4. Methodological Aspects of Model Development

A key methodological decision in the present study was to control for data leakage at the feature selection stage. This point deserves further clarification, as it is often underestimated in studies combining high-dimensional molecular data with machine learning.

If proteins are ranked according to the strength of their association with age using the entire cohort before cross-validation begins, then the samples subsequently assigned to the validation split have already indirectly contributed to the construction of the feature list. In other words, the model receives disguised information about the validation data even before the formal training stage. This leads to systematically optimistic performance estimates that are not reproduced when the model is applied to genuinely new data. In the present study, this source of bias was eliminated: protein ranking and median imputation of missing values were performed exclusively within the training portion of each split. In each case, the model “saw” only the data that would have been available to it in a real-world application scenario.

Within the random-split framework, the 50-feature Gradient Boosting strategy yielded MAE = 8.90 years in repeated cross-validation and MAE = 9.10 years in nested cross-validation. These analyses quantify internal performance after leakage-aware feature selection and model selection. Because they do not separate acquisition batches, they are secondary to the complete-batch-holdout estimates of MAE = 10.56–11.87 years for evaluating generalization.

Multiple configurations were evaluated across four algorithms and several feature counts. Although nested cross-validation partially accounts for configuration-selection bias, no independent external cohort or untouched test set was available. Neither the fitted 50-feature model nor the post hoc fixed eight-protein candidate panel is ready for clinical, diagnostic, or commercial application.

### 3.5. Comparison with Previous Proteomics-Based Age-Prediction Studies

Several previous studies have demonstrated that plasma protein intensity profiles can track chronological age. Lehallier et al. identified nonlinear “waves” of change in the plasma proteome across the human lifespan using the SOMAscan affinity-based platform [9]. Tanaka et al. described a plasma proteomic signature of age based on data generated with the SOMAscan platform [10]. In a more recent study, large-scale proteomic ageing clocks developed using UK Biobank data were independently validated against mortality and common age-associated diseases across several populations [11]. Salignon et al. applied mass-spectrometry-based plasma proteomics in combination with small RNA data and showed that protein-based models can predict chronological age, while the integration of multiple molecular layers improves predictive accuracy [13].

The present study evaluates an exclusively DIA-MS-based workflow in a Moscow cohort of 350 participants across a broad age range. The 222 quantified protein groups reflect the specific non-immunodepleted plasma workflow and should not be interpreted as a comprehensive plasma ageing signature. The principal methodological contribution is a transparent comparison of leakage-aware internal validation with complete-batch holdout under substantial acquisition-design confounding.

### 3.6. Cohort Limitations: Sex and Age Imbalance

The sex imbalance in the older age groups is a central limitation of the study. In the 60–74-year and ≥75-year groups, men were represented by only 15 participants in each group, compared with 68 and 54 women, respectively. The small male strata produced wide confidence intervals, and their estimates should be regarded as exploratory rather than as robust evidence of subgroup-specific model performance.

The higher random-split error in men (MAE = 10.37 years) than in women (MAE = 8.19 years) may reflect the small and imbalanced older male strata, but the present data do not permit a causal or biological interpretation of this difference. It would be inappropriate to conclude that ageing is more predictable in women; the subgroup results are exploratory and may reflect cohort and acquisition structure.

Accordingly, the available data do not support strong conclusions about sex differences in molecular ageing. Menopause-related mechanisms could not be evaluated because the relevant covariates and balanced validation data were unavailable.

The cross-sectional design is another major limitation. A model trained to predict chronological age at a single time point may capture cohort effects, survival patterns, or population structure, but not the individual rate of molecular ageing. Longitudinal data are required to determine whether prediction residuals are associated with future functional decline, disease onset, or mortality.

Chronological age was defined for each sample using the documented date of birth and the individual examination/blood-collection date. The resulting cohort age range was 19.8–99.1 years, with a mean age of 54.4 years. Three discrepancies between sex recorded in the report and clinical-dataset sex codes were identified during data mapping; report sex was used for consistency with the original cohort summary. This source discrepancy does not affect the age calculation.

### 3.7. Reproducibility and Data Availability

This study was conducted in collaboration with a partner research group. Biological samples from 350 participants, stored in our biobank, were transferred to the collaborating group, which performed the mass spectrometry analysis and subsequent processing of the proteomic data. Under the applicable funding conditions and data-governance arrangements, control of the complete proteomic dataset remains with the partner institution. Consequently, the authors of the present study are not currently authorized to independently deposit or distribute the raw or processed proteomic data.

The collaborating research group intends to deposit the relevant data in an appropriate public repository in connection with a separate publication. Once the deposition has been completed, the data will become accessible to the scientific community under the corresponding repository accession number. Prior to their public release, requests for data access may be directed to the corresponding author of the present article and will be forwarded to the data-holding institution. Such requests will be considered by that institution on a case-by-case basis, subject to applicable institutional requirements, informed-consent provisions, and data-sharing agreements. Therefore, the lack of public access to the complete dataset at the time of publication represents a temporary limitation resulting from the allocation of data rights and responsibilities within the collaborative study.

### 3.8. Perspectives for Future Research

An essential next step is external validation. The same analytical workflow should be applied to an independent cohort, preferably measured in a different series of DIA-MS experiments or on a different instrumental platform. This will make it possible to assess the extent to which model performance is determined by a universal biological signal rather than by the specific characteristics of a particular cohort or instrumental workflow.

A cohort balanced by age and sex is needed to enable a proper assessment of model performance across subgroups and to address the limitations described above. The inclusion of additional clinical covariates—body mass index, smoking status, medication use, C-reactive protein, data on comorbidities, and routine biochemical parameters—would help distinguish the signal directly associated with ageing from that driven by inflammation or chronic disease.

Longitudinal profiling is important because molecular ageing is multisystemic and individual-specific [18], whereas a cross-sectional design cannot capture within-person trajectories. Longitudinal data are required to determine whether a proteomics-based chronological-age prediction residual is associated with clinically meaningful outcomes.

Finally, it should be noted that the present study has a limitation related to the level of analysis. The workflow used here operates with aggregate intensities of protein groups and does not provide information on individual proteoforms—variants of the same protein arising from alternative splicing, post-translational modifications, or other molecular events. Given that proteoforms of the same protein may have substantially different biological functions and may change differently during ageing, proteoform-level analysis represents an independent direction for future research.

## 4. Materials and Methods

### 4.1. Study Design and Cohort

The present study used a cross-sectional design and included EDTA-anticoagulated blood plasma samples from 350 adult volunteers obtained from the biobank of the Russian Gerontology Clinical Research Centre of the Pirogov Russian National Research Medical University (Moscow). At the documented examination/blood-collection date, participants ranged in age from 19.8 to 99.1 years (mean ± standard deviation: 54.4 ± 22.9 years). The cohort comprised 231 women and 119 men. Participants were classified into five age groups: 18–24 years (*n* = 64), 25–44 years (*n* = 64), 45–59 years (*n* = 70), 60–74 years (*n* = 83), and ≥75 years (*n* = 69) (Table 4).

According to the original biobank documentation, the cohort was assembled from apparently healthy volunteers. However, detailed clinical covariates—including body mass index, smoking status, medication use, C-reactive protein levels, the presence of cardiovascular risk factors, comorbidities, and subclinical inflammation—were not available for the present analysis. Therefore, throughout this study, the cohort is considered an apparently healthy population with a limited set of covariates, rather than a clinically verified reference group. This distinction is essential for interpreting proteins associated with inflammation and blood coagulation: their associations with age cannot be unequivocally disentangled from the effects of unmeasured clinical factors, which represents an important limitation of the study.

The sex and age structure of the cohort also warrants particular attention. The sex imbalance increased across the age groups: in the 60–74-year group, the female-to-male ratio was 68 to 15, while in the group aged 75 years and older it was 54 to 15. This imbalance should be taken into account when interpreting both predictive performance and the composition of age-associated protein markers.

### 4.2. Sample Preparation

Plasma samples with a volume of 2.5 µL were diluted eightfold with 50 mM triethylammonium bicarbonate buffer (TEAB, pH 8.5). Disulfide bonds were reduced with tris(2-carboxyethyl)phosphine (TCEP; Thermo Fisher Scientific, Waltham, MA, USA) to a final concentration of 40 mM, followed by alkylation with chloroacetamide (CAA; Sigma-Aldrich, St. Louis, MO, USA) to a final concentration of 60 mM. The samples were incubated at 80 °C for 40 min. Proteolytic digestion was performed using sequencing-grade trypsin (Molekta, Moscow, Russia) at an enzyme-to-substrate ratio of 1:25, followed by overnight incubation. The reaction was quenched by adding formic acid to a final concentration of 5%, after which the samples were centrifuged at 14,000× *g* for 20 min at room temperature. The supernatant was dried in a vacuum concentrator at 45 °C, and the resulting peptides were reconstituted in 0.1% formic acid. Immediately before mass spectrometric analysis, stable isotope-labelled synthetic peptide standards were added.

The sample preparation protocol did not include an immunodepletion step for high-abundance plasma proteins. Accordingly, the final matrix comprising 222 proteins reflects the coverage achievable within this standardized workflow and is not intended to provide an exhaustive representation of low-abundance regulatory plasma proteins.

### 4.3. LC–MS/MS Analysis

Chromatographic separation of peptides was performed using an Ultimate 3000 RSLCnano nano-liquid chromatography system (Thermo Fisher Scientific) coupled online to a Q Exactive HFX mass spectrometer (Thermo Fisher Scientific) via a nano-electrospray ionization source. One microgram of the peptide mixture was loaded onto an Acclaim µ-Precolumn trap column (0.5 × 3 mm, 5 µm particle size; Thermo Fisher Scientific) at a flow rate of 10 µL/min for 1 min, using 2% acetonitrile and 0.1% formic acid as the loading solvent. Peptides were separated on a Peaky C18 analytical column (75 µm × 150 mm, 2 µm particle size; Molekta) at a flow rate of 0.3 µL/min. Instrument control and data acquisition were performed using Xcalibur software (version 4.2; Thermo Fisher Scientific). The gradient elution programme was as follows: 2% mobile phase B for 1 min, a linear increase to 35% B over 20 min, an increase to 99% B over 1 min, holding at 99% B for 2 min, a return to 2% B over 1 min, and equilibration at 2% B for 5 min. The total chromatographic run time was approximately 30 min.

The mass-spectrometry measurements were performed using the equipment of “Human Proteome” Core Facilities of the Institute of Biomedical Chemistry (Russia). Mass spectrometric analysis was performed in positive ionization mode on a Q Exactive HFX platform using a standardized variable-window data-independent acquisition scheme and a 30-min chromatographic workflow. The downstream analytical-matrix workflow is described in Section 4.4 and Supplementary Table S11.

The final analytical series comprised 350 biological samples and 350 corresponding RAW files. Each sample was analyzed as a single-shot, single-injection DIA-MS acquisition; there were no technical replicate injections for the same biological sample. Consequently, no technical-replicate aggregation rule was applied in generating the 350-sample analytical matrix.

The augmented matrix records the acquisition order of all 350 samples and assigns them to 27 MS batches. No experimenter-side batch-effect correction or downstream transformation was applied to the final protein–group matrix before statistical analysis. The processing log and augmented matrix do not contain quantitative summaries of instrumental drift, quality-control sample trajectories, or carryover; these quantities therefore could not be reconstructed retrospectively and remain a limitation of the study.

### 4.4. DIA Data Processing and Protein Quantification

The final protein–group matrix was generated with DIA-NN version 1.8.1 using a two-pass predicted-library-assisted workflow. The starting spectral library, Human3_library.predicted.speclib, had been generated in silico from uniprot_Human_2024_02_13.fasta; the DIA-NN processing log records reannotation against uniprotkb_Human_2025_03_13.fasta before the final DIA-NN analysis. Tryptic cleavage with one allowed missed cleavage was specified, with peptide lengths of 7–30 amino acids, precursor m/z 300–1800, precursor charges 1–4, and fixed carbamidomethylation of cysteine. Mass-accuracy settings were 10 ppm for MS2 fragment ions and 20 ppm for MS1 precursor ions. The processing workflow comprised library generation followed by reanalysis, q-value 0.01, relaxed protein inference, smart profiling, peak-centre refinement, and output-matrix generation. The final protein–group matrix was filtered by DIA-NN at 1% precursor and 1% protein–group FDR. Cross-run precursor-level normalization remained enabled, and protein–group quantities were generated using the DIA-NN normalized MaxLFQ-like procedure. No additional transformation or filtering was applied to the exported table before statistical analysis. The final matrix contained semi-quantitative label-free intensities for 222 protein groups across 350 biological samples.

Among the included features, one was completely absent in at least one training split during cross-validation. In such cases, the feature was excluded from the corresponding split; no imputation based on information from the validation subset was performed. In all other cases, missing values were imputed using the median intensity calculated exclusively from the training data of the respective split.

### 4.5. Calculation of Chronological Age

Chronological age was calculated as the difference between the date of blood collection and the date of birth.

### 4.6. Statistical Analysis and Machine Learning

All calculations were performed in Python 3.12 using the pandas, NumPy, SciPy, and scikit-learn libraries. A key requirement of the analytical protocol was to prevent any leakage of information from the validation splits into the feature selection stage: proteins were ranked according to their association with age strictly within each training subset, without using information from the corresponding validation subset in any form.

#### 4.6.1. Descriptive Correlation Analysis of Protein–Age Associations

To characterize age-associated plasma proteins across the entire cohort, Spearman rank correlation coefficients and Pearson correlation coefficients were calculated between the intensity of each protein and chronological age. Statistical significance was assessed at α = 0.05 with Bonferroni correction for multiple comparisons (222 tests; adjusted significance threshold, *p* < 2.25 × 10^−4^). The results of this analysis are descriptive and reflect the age-associated structure of the cohort proteome; they were not used as a feature-selection criterion for the predictive models.

#### 4.6.2. Leakage-Aware Feature Selection

When building the predictive models, protein ranking was performed separately within each training split. For each split, missing values were imputed using the median calculated from the training data; proteins were then ranked by the absolute value of their Spearman correlation coefficient with chronological age, and a predefined number of the strongest predictors was selected (5, 8, 10, 12, 15, 20, 30, or 50). The validation split was not used at any stage of feature ranking or feature selection. This approach prevents optimistic bias in performance estimates that can arise when features are ranked globally across the full cohort before cross-validation.

#### 4.6.3. Model Development and Evaluation

Four regression algorithms were compared: ridge regression, Elastic Net, Random Forest, and Gradient Boosting. Ridge regression and Elastic Net were applied with feature normalization incorporated within the analytical pipeline. The Random Forest model was built using 100 trees with a maximum depth of 8. Gradient Boosting used 200 base estimators, a maximum tree depth of 4, a learning rate of 0.1, and a fixed random seed (random state = 42).

Model evaluation comprised complementary analyses with distinct purposes. Repeated five-fold cross-validation with three repetitions compared algorithms and feature-number strategies; feature selection was repeated within every training fold, and the reported Gradient Boosting result (MAE = 8.90 ± 1.06 years) is the mean ± standard deviation of 15 fold-level MAEs. Nested five-by-three-fold cross-validation assessed uncertainty in algorithm and feature-count selection (MAE = 9.10 ± 1.10 years). A separate predefined five-fold OOF vector supported subgroup summaries (aggregate MAE = 8.93 years), and a matched random five-fold sensitivity analysis yielded MAE = 8.92 years. Because these random-split analyses can share acquisition structure across training and test folds, complete-batch-holdout analyses described in Section 4.6.6 provide the primary estimate of generalization. Table 4 summarizes the role and metrics of each analysis.

#### 4.6.4. Evaluation of Reduced Feature-Number Strategies

Strategies using 5, 8, 10, 12, 15, 20, 30, and 50 features were evaluated. Protein ranking was repeated within each training split, and all four algorithms were tested at each feature count. Consequently, reported cross-validation metrics apply to the strategy of selecting a given number of proteins within each training fold, not to any fixed post hoc panel. The fixed eight-protein list was derived from full-cohort descriptive ranking after model comparison and is reported only as an exploratory candidate panel for future independent validation.

#### 4.6.5. Sensitivity Analyses by Sex and Age

To assess sensitivity to the uneven sex and age distribution, sex-adjusted protein–age correlations were calculated from regression residuals. Out-of-fold predictions from the 50-feature Gradient Boosting analysis were summarized by sex and age group using *n*, MAE, mean and median prediction residuals, and nonparametric bootstrap 95% confidence intervals based on 10,000 resamples. R^2^ was retained for the overall cohort and sex strata but omitted for narrow age strata because the restricted outcome range makes it unstable and poorly interpretable. These analyses assess demographic sensitivity and do not establish biological differences in ageing between sexes.

#### 4.6.6. Sensitivity Analyses for Acquisition Order and MS Batch

The augmented matrix was linked to the exact collection-date-derived chronological ages and used to assess acquisition-design confounding. Association between age and acquisition order was tested by Spearman correlation; age differences across the 27 batches were tested by the Kruskal–Wallis test; and sex distribution across batches was tested by Pearson’s chi-square test. Metadata-only Gradient Boosting negative controls were evaluated in the same random five-fold split using order alone, batch alone, and order, batch, and sex together. For the protein model, a matched random five-fold analysis was compared with two batch-held-out schemes: standard GroupKFold and a deterministic stratified assignment of complete batches to five folds to reduce fold-size and age-distribution imbalance. All samples from a given MS batch remained together in the held-out fold.

Residuals from the matched random-split protein model were tested against acquisition order and batch. Principal-component analysis was performed on log2-transformed protein intensities after median imputation and standardization; batch effect size for PC1 and PC2 was summarized by η^2^, and component scores were compared to order by Spearman correlation. As a conservative sensitivity analysis, partial rank correlations were estimated after adjustment for sex, order, and batch, and a foldwise nuisance-residualized protein model was evaluated. Because chronological age was itself strongly associated with order and batch, the adjusted analyses were interpreted only as sensitivity checks and not as unbiased estimates of biological age effects.

## 5. Conclusions

The present study demonstrates that DIA-MS-based plasma proteomics can identify age-associated protein patterns and support proof-of-concept chronological-age prediction in a Moscow cohort of 350 participants.

Descriptive correlation analysis identified 148 proteins significantly associated with chronological age at a nominal threshold of *p* < 0.05, of which 98 remained significant after Bonferroni correction. The strongest associations were observed for proteins involved in inflammation, blood coagulation, activation of the complement system, oxidative stress, and heme metabolism. Overall, the resulting protein signature is consistent with the concept of inflammageing; however, in the absence of detailed clinical covariates, it is not possible to clearly distinguish the contribution of chronological ageing from that of unmeasured factors, such as subclinical inflammation, comorbidities, and lifestyle.

With feature selection confined to each training subset, internal random-split analyses yielded MAE = 8.90 years in repeated cross-validation and MAE = 9.10 years in nested cross-validation. More importantly, complete-batch holdout yielded MAE = 10.56–11.87 years and therefore provides the primary estimate of generalization from the present dataset. The 8-feature strategy yielded MAE = 11.83 years in repeated random cross-validation; the specific fixed eight-protein list remains a post hoc exploratory candidate set and has not been independently validated.

The model predicts chronological rather than biological age, has not undergone external validation, and has not been tested against mortality, functional decline, disease onset, or longitudinal ageing trajectories. The cohort is imbalanced by sex and age, detailed clinical and lifestyle covariates were unavailable, and the 222-protein matrix does not represent comprehensive plasma–proteome coverage. Most importantly, age and sex were strongly structured by acquisition order and MS batch. The findings should therefore be regarded as proof-of-concept evidence within this dataset. Independent validation requires a cohort prospectively randomized or balanced across age, sex, run order, and batch, with predefined quality-control procedures and clinically meaningful outcomes.

## Figures and Tables

**Table 1:** Top 10 Descriptive Age-Correlated Proteins.

Rank	Protein ID	Protein Name	Spearman rs	Biological Theme	Principal Function
1	FIBB	Fibrinogen beta chain (FGB)	0.590	Coagulation/inflammation	Fibrinogen subunit involved in fibrin clot formation and acute-phase responses
2	AACT	Alpha-1-antichymotrypsin/ SERPINA3	0.560	Acute-phase response	Serine protease inhibitor involved in acute-phase and inflammatory responses
3	ITIH4	Inter-alpha-trypsin inhibitor heavy chain 4	0.542	Inflammation/ECM	Acute-phase glycoprotein involved in extracellular-matrix stabilization
4	CO5	Complement C5	0.538	Complement cascade	Complement component cleaved to C5a/C5b during terminal-pathway activation
5	CERU	Ceruloplasmin (CP)	0.518	Oxidative stress/metal metabolism	Copper transport and ferroxidase activity; contributes to redox homeostasis
6	C4BPA	C4b-binding protein alpha	0.518	Complement regulation	Negative regulator of the classical and lectin complement pathways
7	AMBP	Alpha-1-microglobulin/bikunin precursor	0.513	Inflammation/heme handling	Precursor of alpha-1-microglobulin and bikunin; heme scavenging and protease inhibition
8	HEMO	Hemopexin (HPX)	0.513	Heme metabolism	High-affinity heme scavenging and protection from heme-mediated oxidative injury
9	A1BG	Alpha-1B-glycoprotein	0.495	Plasma glycoprotein	Circulating glycoprotein with incompletely defined physiological function
10	FIBG	Fibrinogen gamma chain (FGG)	0.475	Coagulation	Fibrinogen subunit involved in clot formation and platelet aggregation

**Table 2:** Cross-Validated Accuracy of Gradient Boosting Feature-Number Strategies.

Number of Fold-Selected Features	MAE, Years	R^2^
5	13.38	0.375
8	11.83	0.502
10	11.62	0.518
12	11.27	0.544
15	10.96	0.574
20	10.30	0.595
30	9.27	0.670
50	8.90	0.698

**Table 3:** Summary and interpretation of validation analyses.

Validation Strategy	Folds/Repetitions	Feature Selection	Nested Model Selection	Analytical Role	MAE, Years	R^2^
Repeated random CV	5/3	Within fold	No	Internal comparison	8.90 ± 1.06	0.698 ± 0.078
Nested random CV	5 outer/3 inner	Within fold	Yes	Internal selection check	9.10 ± 1.10	0.680 ± 0.086
Predefined random OOF	5/1	Within fold	No	Subgroup summaries	8.93	0.704
Matched random sensitivity	5/1	Within fold	No	Batch-holdout comparator	8.92	0.699
Batch-stratified holdout	5/1	Within fold	No	Primary estimate	10.56	0.554
GroupKFold batch holdout	5/1	Within fold	No	Conservative sensitivity	11.87	0.461

**Table 4:** Cohort demographics by age group and sex.

Age Group	Total	Women	Men	Women %
18–24	64	35	29	54.7
25–44	64	36	28	56.3
45–59	70	38	32	54.3
60–74	83	68	15	81.9
75+	69	54	15	78.3
Total	350	231	119	66.0

Clinical-data availability: detailed diagnoses, body mass index, smoking status, medication use, C-reactive protein, cardiovascular risk factors, comorbidities, and subclinical inflammation were not available for this analysis; the cohort cannot be treated as a clinically verified disease-free reference population.

## Data Availability

Complete feature-level correlation, feature-stability, cross-validation, batch-sensitivity, and DIA-processing outputs supporting the reported results are provided in Supplementary Tables S1–S14. The complete processed protein matrix and raw DIA-MS files are controlled by the collaborating institution under the applicable funding and data-governance arrangements and cannot currently be deposited or distributed independently by the authors. Requests may be directed to the corresponding author and will be forwarded to the data-holding institution for case-by-case consideration under institutional, informed-consent, and data-sharing requirements. The collaborating group intends to deposit the relevant data in a public repository with a separate publication; the accession number is not yet available.

## Supplementary Materials

The following supporting information can be downloaded at: https://www.mdpi.com/article/10.3390/ijms27198707/s1.

## Abbreviations

The following abbreviations are used in this manuscript:

DIA-MS	Data-independent acquisition mass spectrometry
LC-MS/MS	Liquid chromatography–tandem mass spectrometry
DIA-NN	Data-independent acquisition by neural networks
MAE	Mean absolute error
R^2^	Coefficient of determination
EDTA	Ethylenediaminetetraacetic acid
TEAB	Triethylammonium bicarbonate
TCEP	Tris(2-carboxyethyl)phosphine
CAA	Chloroacetamide
AGC	Automatic gain control
ECM	Extracellular matrix
BMI	Body mass index
CRP	C-reactive protein
FGB	Fibrinogen beta chain
FGG	Fibrinogen gamma chain
SERPINA3	Alpha-1-antichymotrypsin
ITIH4	Inter-alpha-trypsin inhibitor heavy chain H4
C5	Complement component C5
C4BPA	C4b-binding protein alpha chain
CP	Ceruloplasmin
AMBP	Alpha-1-microglobulin/bikunin precursor
HPX	Hemopexin
A1BG	Alpha-1B-glycoprotein
ZA2G	Zinc-alpha-2-glycoprotein
PRIDE	Proteomics Identifications Database
ProteomeXchange	Proteomics data exchange consortium
HUPO	Human Proteome Organization
CV	Cross-validation
OOF	Out-of-fold
FDR	False discovery rate
PCA	Principal-component analysis
CI	Confidence interval
GB	Gradient Boosting
